# Translational Aspects of Sphingolipid Metabolism in Renal Disorders

**DOI:** 10.3390/ijms18122528

**Published:** 2017-11-25

**Authors:** Alaa Abou Daher, Tatiana El Jalkh, Assaad A. Eid, Alessia Fornoni, Brian Marples, Youssef H. Zeidan

**Affiliations:** 1 Department of Anatomy, Cell Biology and Physiology, Faculty of Medicine, American University of Beirut, Beirut 1107 2020, Lebanon; ara32@mail.aub.edu (A.A.D.); tj09@aub.edu.lb (T.E.J.); ae49@aub.edu.lb (A.A.E.); 2 Department of Medicine, Peggy and Harold Katz Family Drug Discovery Center, University of Miami, Miami, FL 33136, USA; AFornoni@med.miami.edu; 3 Department of Radiation Oncology, Miller School of Medicine/Sylvester Cancer Center, University of Miami, Miami, FL 33136, USA; brian.marples@beaumont.org; 4 Department of Radiation Oncology, American University of Beirut Medical Center, Beirut 1107 2020, Lebanon

**Keywords:** sphingolipids, Fabry’s disease, sphingolipid metabolism, podocytes, renal injury, renal failure

## Abstract

Sphingolipids, long thought to be passive components of biological membranes with merely a structural role, have proved throughout the past decade to be major players in the pathogenesis of many human diseases. The study and characterization of several genetic disorders like Fabry’s and Tay Sachs, where sphingolipid metabolism is disrupted, leading to a systemic array of clinical symptoms, have indeed helped elucidate and appreciate the importance of sphingolipids and their metabolites as active signaling molecules. In addition to being involved in dynamic cellular processes like apoptosis, senescence and differentiation, sphingolipids are implicated in critical physiological functions such as immune responses and pathophysiological conditions like inflammation and insulin resistance. Interestingly, the kidneys are among the most sensitive organ systems to sphingolipid alterations, rendering these molecules and the enzymes involved in their metabolism, promising therapeutic targets for numerous nephropathic complications that stand behind podocyte injury and renal failure.

## 1. Podocytes

### 1.1. Renal Glomerular Podocyte Role and Function

Podocytes are highly differentiated visceral epithelial cells in the glomerulus of the kidney. They are similar to neurons in that they are always in a quiescent state and thus do not actively proliferate. Their unique shape and location render them critical for glomerular basic functions such as filtration [1]. While the voluminous cell body of the podocyte incurves into the urinary space of the capsule, its foot processes extend to interdigitate with foot processes from adjacent cells. The proper interaction of foot processes the glomerular basement membrane (GBM) represents a key element of the glomerular filtration barrier. These specialized processes interdigitate to form filtration slits, which allow only small molecules to pass from the blood into the first filtrate [2].

### 1.2. Podocyte Injury

Podocyte integrity is crucial for maintaining the stability of the filtration barrier. Insults to the glomerular podocytes from numerous sources including metabolic cellular stress, genetic mutations, cancer and inflammatory, metabolic and hemodynamic changes usually result in functional aberrations [3]. Podocyte dysfunction is characterized by cytoskeletal remodeling or dysregulation and foot processes widening, effacement and loss. These structural changes eventually compromise the filtration barrier and large, negatively charged molecules such as proteins are able to cross from the blood into the urine. The latter process is referred to as proteinuria, and is one of the first signs of glomerular diseases [4].

## 2. Sphingolipids

Little attention has been given in biomedical research to the role of lipids in the pathogenesis of podocyte dysfunction. In particular, sphingolipids were long thought to be passive spectator barrier lipids in cell membranes. Recently, researchers have started to appreciate the implication of sphingolipids in podocyte injury. To set the stage for discussion of the role of sphingolipids in the pathophysiology of podocyte-related problems, we will briefly describe their metabolism and shed light on the major discoveries that highlight the involvement of sphingolipids in health and disease states.

### 2.1. Overview of Sphingolipid Metabolism

Sphingolipids are a class of ubiquitous lipids that share a sphingosine base backbone and comprise more than a thousand naturally occurring molecules. Sphingolipids constitute an essential part of eukaryotic cell membranes and are known to regulate the fluidity and subdomain structure of the cell membrane lipid bilayer [5,6]. Beyond their identified structural role, sphingolipid metabolism has proved to be part of an extensive network of regulated signaling pathways that produce bioactive molecules involved in several fundamental cellular processes such as proliferation, differentiation, senescence and cell-to-cell interactions [7,8,9,10]. These bioactive molecules are mainly ceramide, sphingosine, sphingosine-1-phosphate (S1P) and ceramide-1-phosphate (C1P), among many others.

Several contemporary chemical and biotechnological techniques have been applied to the study on sphingolipids and the implications of sphingolipid dysregulation, along with their metabolic pathways in the pathophysiology of cancer, angiogenesis, atherosclerosis, inflammation, insulin resistance and others. Remarkably, sphingolipids are being increasingly implicated in human diseases that contribute to podocyte dysfunction, including diabetes and various metabolic disorders. For more details on the role of sphingolipids in disease states, the reader can refer to a set of well-written reviews [11,12,13,14].

Ceramides share a sphingoid base attached to a fatty acid. These molecules are structural precursors and are central for the metabolism of other sphingolipids. Different ceramides, thought to play distinct roles in cellular pathways, are characterized by the degree of saturation and length of their attached fatty acyl chain, and are the products of different biochemical reactions of different enzymes [15,16,17,18,19,20].

The biosynthesis of ceramide usually takes place in the endoplasmic reticulum or the plasma membrane, at baseline or upon exposure of cells to a stressor such as heat, radiation, chemotherapy or hypoxia [17,21,22,23,24], and can occur through three distinct pathways, one of which is de novo synthesis. De novo synthesis starts through the condensation on l-serine and palmitoyl-coA, yielding 3-ketosphinganine. This reaction occurs on the surface of the intracellular endoplasmic reticulum, catalyzed by the enzyme serine-palmitoyl transferase (SPT). 3-Ketosphinganine is subsequently reduced to sphinganine. A reaction between sphinganine and a fatty acyl coA, catalyzed by ceramide synthase (CerS), yields dihydroceramide, which is then dehydrogenated to give ceramide. The products of this chain of reactions usually remain anchored to the endoplasmic reticulum surface [25,26,27] until the produced ceramide is transported to other subcellular compartments, such as the Golgi apparatus, where it can be metabolized to generate other sphingolipids [21,28,29]. CerS also catalyzes the production of ceramide from sphingosine by the salvage pathway, through which long-chain fatty acids are recycled. Six isoforms of CerS are encoded by six different genes of the same family, each of which produces a distinct ceramide species unique in its fatty acid composition and degree of saturation [30,31,32,33,34,35]. Finally, ceramide can also be produced from the hydrolysis of sphingomyelin (SM) or other complex sphingolipids by sphingomyelinases. Phosphocholine is produced as a byproduct of this hydrolysis reaction [36].

Ceramidase, an enzyme that breaks down the amide bonds of the ceramide molecule, generates sphingosine and free fatty acids. Three different ceramidases have been described in the literature, each of which functions at an optimum pH [37,38,39,40,41,42,43,44,45,46,47,48,49,50]. Sphingosine is an important signaling molecule that principally functions to halt the progression of the cell cycle and induce apoptosis. Just like ceramides, the effects of sphingosine are mediated through several intracellular enzymes, including protein kinases and phosphatases [36,51,52]. This is explained further in a review by Pettus et al. [53].

Sphingosine-1-phosphate (S1P) can be formed from sphingosine by the action of sphingosine kinase. It acts a ligand for a set of G-protein couples receptors (GPCRs) and can exert some receptor-independent intracellular effects as well [54,55]. Contrary to the observed effects of ceramides, which might be classified as tumor-suppressive lipids, S1P has been implicated in cellular growth, survival and migration, and was found to play a role in angiogenesis and immune responses [56]. Sphingosine-1-phosphate can be deactivated by the activity of either S1P phosphatase or S1P lyase [57]. Interestingly, mice with podocyte-specific deletion of S1P lyase develop proteinuria [58] and S1P lyase genetic mutation results in severe podocyte injury and proteinuria [59].

Ceramide kinase (CERK) is an enzyme that catalyzes the formation of ceramide phosphate (C1P), and is yet another bioactive phosphorylated sphingolipid thought to play an extensive role in inflammation [60]. In fact, C1P has been implicated in eicosanoid production; it recruits the enzyme responsible for arachidonate release to the plasma membrane, where arachidonate, the precursor of eicosanoids resides and stimulates its activity [61,62]. Conversely, some evidence suggests that C1P acts as an anti-inflammatory molecule by inhibiting the enzyme that converts pro-TNF (tumor necrosis factor) to its active inflammation-inducing form [63], thus halting the production of TNF. Indeed, the downregulation of CERK in mouse models of airway hyper-responsiveness using silencing ribonucleic acid (RNA) molecules lead to a significant decrease in the observed inflammatory response [64]. In addition, other studies have shown that exogenous administration of C1P can inhibit the production of several interleukins including IL-6, IL-8 and IL-1β from peripheral blood mononuclear cells [65]. Given this controversial data, the concept of using this newly identified bioactive sphingolipid in immunomodulation remains debatable and requires further research to reveal its intricately complex systemic effects. Interestingly though, despite the ongoing investigations regarding C1P and all its physiological roles, a cell-surface C1P receptor through which circulating C1P can act has not been identified. Similarly, the identity of a C1P lyase was suggested but remains unknown.

### 2.2. Sphingolipids in Health and Disease

#### 2.2.1. Sphingolipids and Cancer Treatment

Sphingolipids are being extensively studied as potential therapeutic targets in cancer research. Ceramides, for example, have been recognized as tumor-suppressive lipids that exert anti-proliferative effects including growth arrest and apoptosis [13,66]. Moreover, earlier studies had shown that short-chain ceramides induce apoptosis in several cancer cell types [67]. In addition, the inclusion a mixture of short-chain ceramides in the treatment of breast cancer patients improved symptom control with only minimum side effects reported [68]. The increased expression of some ceramidases has also been observed in several treatment-resistant prostate cancer specimens and cell lines [69], and the inhibition of ceramidase activity proved to have an anticancer effect. This, if anything, hints at a promising role for sphingolipids and their metabolic pathway in cancer therapy.

Drug resistance is a major concern in cancer treatment. In fact, upon exposure to stressors, some cancer cell types alter sphingolipid metabolism and become drug resistant, which promotes tumor growth and survival [70,71]. For instance, earlier experiments have demonstrated that glucosylceramide synthase (GCS) is capable of modulating drug resistance. GCS is an enzyme involved in sphingolipid metabolism that transfers glucose from UDP-glucose (uridine diphosphate- glucose) to ceramide producing glucosylceramides, and its implications in the development of cancer drug resistance have garnered considerable interest in the past few years [72,73,74,75,76]. Several mechanisms have been proposed through which the cell can alter the cytotoxicity of the chemotherapeutic agents used, and many of those mechanisms involve modulation of GCS expression. GCS is known to catalyze the first step in the production of glycosphingolipids [77,78], which are important players in tumor progression. Their dysregulation has been extensively linked to the angiogenesis and metastasis of chemoresistant cells. Glycosphingolipids include lactosylceramides, gangliosides and glucosylceramides. Indeed, blocking membrane ganglioside synthesis by fumonisin B1 enhanced the radiosensivity of human melanoma cells [79].

Experiments by Liu et al. demonstrated that transfecting MCF-7 resistant cells with GCS antisense resensitized the cells to doxorubicin, vinblastine and paclitaxel. Multi-drug resistance (MDR) is a property of cancerous cells conferred by members of the ABC (ATP-binding cassette) transporters family. These are membrane transporters, some of which encoded by the MDR1 gene, that reduce the intracellular availability and cytotoxicity of anti-cancer drugs through efflux outside the cell [80]. In fact, a study conducted by Gouaze et al. [80] showed that transfecting resistant human breast cancer cells with GCS antisense reduced the expression of the MDR1 gene and altered the phospholipid bilayer composition in such a way that enhanced the sensitivity of the cells to the used drug. Therefore, GCS is a potential target in chemoresistant cancer cells.

Several lines of evidence support the involvement of S1P in tumor growth and survival [62,63,64]. Visentin et al. [81] used S1P monoclonal antibodies to slow down the progression and even eliminate human and mice tumors in in vivo and in vitro models, thus demonstrating the pro-oncogenic role of S1P. Other lines of evidence support that VEGF (vascular endothelial growth factor) receptors are transactivated by S1P, and thus implicate a role for S1P in the vasculature of the tumor, which is important for the maintenance of tumor microenvironment, metastasis and invasion [81,82]. S1P exerts its functions through a family of G-protein coupled receptors S1P_1–5_. In addition to regulating vascular maturation and angiogenesis, it has been implicated in many aspects of cancerous cells such as cytoskeletal arrangement and movement, calcium homeostasis, and cellular growth and inhibition of apoptosis [82]. Thus, it is not surprising that sphingosine kinase inhibitors are currently in clinical trials for the treatment of solid tumors.

#### 2.2.2. Sphingolipids and Inflammatory Responses

Inflammation is the body’s natural protective response against foreign infectious agents and allergens. Recent literature hints at the implication of sphingolipids, namely C1P and S1P, in inflammatory responses by activating mediators such as prostaglandins. C1P has been shown to regulate the activation and the translocation of cytosolic phospholipase A_2_ (cPLA2), in a calcium-dependent manner, in response to inflammatory responses such as Interleukin-1β (IL1β) and tumor necrosis factor α (TNF-α), thus leading to the production of prostanoids from arachidonic acid. This latter step requires the activation of cyclooxygenase-2 (COX-2), which is mediated by S1P. Unlike the mechanism of action of C1P, which involves direct binding to specific domains of PLA2, S1P has been shown to act indirectly to stimulate COX-2 activity by modulating its expression at the messenger RNA (mRNA) level [9,61,83,84]. Taken together, these studies suggest a scheme of events in which C1P and S1P act synergistically in a spatially and temporally coordinated manner to induce eicosanoid production, thereby mounting an orchestrated inflammatory response [85]. In addition to their proinflammatory role in the production of eicosanoids, C1P and S1P are also involved in allergic responses and are crucial for macrophage survival, neutrophil activity and cytokine production [86,87,88,89]. This makes these endogenous sphingolipids and the enzymes responsible for their production, ceramide kinase (CK) and sphingosine kinase (SK), respectively, potential targets for the development of new anti-inflammatory compounds, especially after some COX-2 inhibitors have been withdrawn from the market for their dangerous side effects [84,89,90]. Indeed, interfering with S1P signaling has proven through clinical trials to be effective in targeting autoimmune conditions such as multiple sclerosis [91].

#### 2.2.3. Sphingolipids and Insulin Resistance

Insulin resistance is a hallmark of type 2 diabetes, a metabolic disorder characterized by chronic hyperglycemia and severe complications including neuropathy, retinopathy and diabetic nephropathy, altogether referred to as diabetic triopathy. There is increasing evidence that supports the involvement of sphingolipids in the pathophysiology of insulin resistance. Studies conducted by Shimabukuro et al. showed increased ceramide levels and higher number of apoptotic cells in pancreatic islets isolated from Zucker diabetic rats [92]. This study among others helped establish the concept of cell death from lipid oversupply, also known as lipoapoptosis [93].

Liver and skeletal muscles are important glucose storage organs and, hence, the loss of their sensitivity to insulin promotes the development of type 2 diabetes. Old and current data strongly suggests the involvement of ceramides in insulin resistance. Turinsky et al. reported elevated ceramide levels in liver and skeletal muscles in rat models of insulin resistance. Increased ceramide levels have also been reported in more recent studies in muscle biopsies from subjects displaying risk factors for insulin resistance and from insulin-resistant obese humans [94,95,96]. Additionally, analogs of ceramide or agents that stimulate endogenous ceramides biosynthesis have proved to inhibit systemic insulin signaling effects such as glycogen synthesis and glucose uptake [97,98,99,100].

Although the mechanistic pathway of lipid accumulation in insulin resistance might not be clear yet, it is suggested that there are alterations in the way through which Akt, a serine/threonine protein kinase, responds to ceramide [98]. In fact, research conducted by Summers et al. revealed that ceramide can antagonize the effect of insulin by dephosphorylating and hence inactivating Akt and its downstream effector protein kinase B (PKB) [98,101,102]. Akt acts downstream of insulin and mediates its anabolic effects. Akt/PKB has been implicated in cellular responses to several hormones and growth factors. Signals from these chemicals activate a well-known pathway that starts with the enzyme phosphatidylinositol 3-kinase. The latter is a regulatory lipid kinase that catalyzes the production of phosphatidylinositol 3,4-bisphosphate and phosphatidylinositol 3,4,5-triphosphate. rrAt the plasma membrane, these two phosphoinositides serve as docking sites for Akt/PKB and phosphoinositide-dependent kinase-1 (PDK1) and, hence, gets these kinases closer to each other, activating the recruited isoform of Akt/PKB at its phosphorylating sites and triggering a cascade that elicits the desired cellular response [102]. Indeed, artificially doubling ceramide levels in some insulin-sensitive cells such as preadipocytes and myotubes to mimic diabetic conditions blocks this critical step in insulin response at two different sites in the Akt/PKB complex, through independent mechanisms, thus supporting the proposed cellular mode of response to ceramide accumulation [97,98,103]. Interestingly, podocytes are insulin sensitive cells [104] and podocyte specific deletion of the insulin receptor results in altered AKT phosphorylation and podocyte injury [105,106] However, whether sphingolipids interfere with podocyte insulin signaling remains to be established. For more detailed information on sphingolipids and insulin resistance, the reader can refer to this article [107].

## 3. Sphingolipids in Podocyte Injury

In the past few years, it has become clear that sphingolipids and their metabolic pathways are heavily implicated in podocyte injury, however, the underlying molecular pathways and mechanisms of lipid-mediated kidney and glomerular diseases are still poorly understood. The study and characterization of genetic glycosphingolipid metabolic and storage diseases through several experimental and clinical models have indeed increased the interest in sphingolipid metabolites as therapeutic targets for many non-genetically acquired nephropathic complications.

### 3.1. Globotriaosylceramide (Gb3)

Fabry’s disease (OMIM # 301500) is an X-linked sphingolipidose characterized by deficient activity of a lysosomal hydrolase encoded by the *GLA* gene, α-galactosidase A. The disease phenotype is a result of the intracellular and extracellular build-up of non-metabolized glycosphingolipids. This condition leads to the progressive deposition of the α-galactosidase A substrate, Globotriaosylceramide (Gb3), in virtually all the patient’s tissues. Although end-stage renal disease is one of the leading causes of death in hemizygous males with this inborn error, the mechanism of kidney failure is not well understood. However, based on histological studies, the accumulation of the metabolite Gb3 in the podocytes has been theorized to explain the pathophysiology of the resulting glomerular damage. In the kidneys, podocytes accumulate Gb3 more than all the other cell types leading to podocyte injury that occurs at an early age and eventual podocyturia, where podocytes detach and can be found in the patient’s urine [108,109,110,111].

Due to the absence of appropriate human and animal models to test the hypothesized mechanism, Liebau and colleagues designed a cellular model of Fabry’s disease in which RNA interference and lentiviral transduction techniques were used to knockdown the *GLA* gene from human podocytes. The double deletion of this gene resulted in a decreased α-galactosidase A enzymatic activity and a slow accumulation of intracellular Gb3. Remarkably, the upregulation of LC3-II and the downregulation of mTOR kinase activity, an autophagy inhibitor, were observed. An increase of autophagosomes was also noted as a result of these two changes. The data obtained indicates a link between α-galactosidase A dysregulation and autophagy pathways and hints at promising future directions in uncovering the mechanism of nephropathy in Fabry’s disease to develop an optimal therapy [109,110].

**Figure 1:**
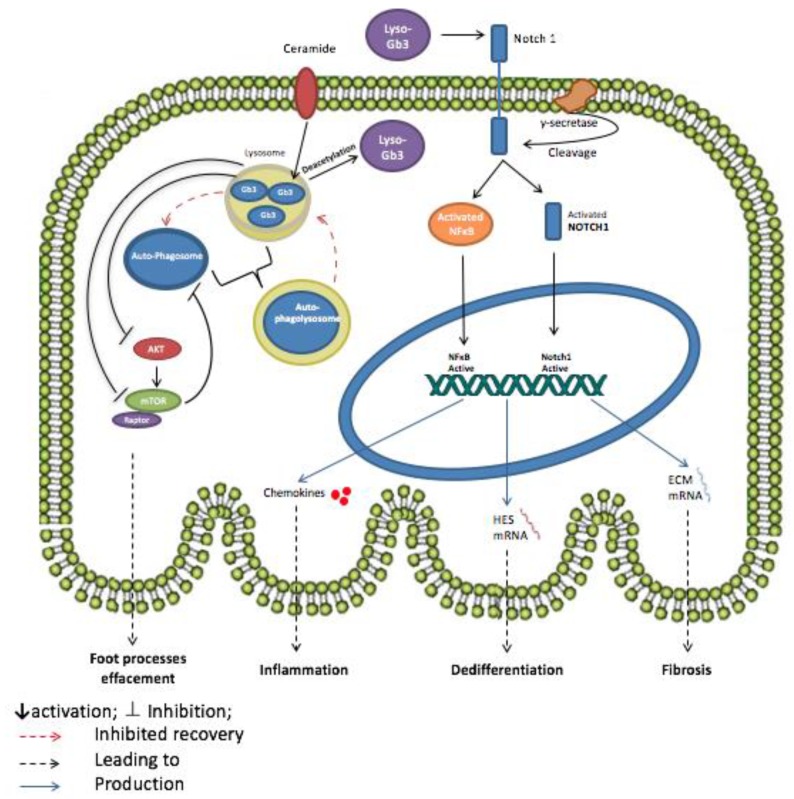
Potential mechanism of action of Globotriaosylceramide (Gb3) and Globotriaosylsphingosine (Lyso-Gb3) in Fabry’s disease podocyte. The accumulation of Gb3 in lysosomes inhibits AKT and mTOR pathway leading to the dysregulation of autophagy signaling in the podocyte. The inhibition of mTOR prevents the recovery of autophagosomes and lysosomes from autophagolysosomes by negative regulation. The formation of the autophagolysosomes causes podocyte foot processes effacement thus injury. Lyso-Gb3, the deacetylated form of Gb3, also plays a role in podocyte injury by promoting inflammation, fibrosis and dedifferentiation of podocytes. Lyso-Gb3 activates both the NOTCH signaling pathway, through γ-secretase, and nuclear factor κB (NFκB) leading to the release of chemokines. NOTCH-1 activation also promotes the transcription of *HES* genes and genes coding for extracellular matrix (ECM) proteins thus inducing, respectively, the dedifferentiation and fibrosis of the podocyte.

Currently, enzyme replacement therapy (ERT) is being clinically used in the management of Fabry’s disease [112,113,114]. However, the onset of the disease occurs during childhood whereas diagnosis is often left until a life-threatening condition develops in the heart, kidneys or nervous system. This time gap between the development of early symptoms and diagnosis and treatment allows enough room for irreversible advanced tissue damage that ERT cannot halt. For example, ERT did not prove to be efficient in improving patient outcomes after the onset of urinary albuminuria, a hallmark of podocyte injury [115], especially in the absence of nephroprotective therapies [116]. Globotriaosylsphingosine (Lyso-Gb3), the deacetylated form of Globotriaosylceramide, is a circulating bioactive glycolipid that has been recently described to increase remarkably in the body fluids of Fabry’s disease patients, such as plasma and urine [117]. In experiments conducted by Sanchez-Nino et al. in an attempt to find a better therapeutic approach to Fabry’s disease, high levels of the Lyso-Gb3 proved to play a proinflammatory role in cultured human podocytes, mainly through the activation of NOTCH-1 signaling pathway [110]. Upon the binding of Lyso-Gb3 to the proper receptor, Notch undergoes a series of proteolytic cleavages and Notch intracellular domain (NICD), a cytoplasmic protein, is produced through the action of γ-secretase. Lyso-Gb3 has also been found to upregulate the expression of *NOTCH-1* in podocytes, which in turn has been shown to lead to kidney fibrosis and cause podocyte injury in vivo. Notch-1 recruits the transcription nuclear factor κB (NFκB), a well-known regulator of inflammatory responses [118], hence increasing its DNA-binding activity. Furthermore, NICD translocates to the nucleus and promotes the expression of NOTCH canonical targets such as the *HES1* gene (hairy and enhancer of split 1), thus promoting dedifferentiation of the podocytes, genes coding extracellular matrix proteins such as fibronectin, thus leading to fibrogenic responses, and inflammatory genes such as chemokines, resulting in a state of inflammation. Indeed, siRNA silencing of NOTCH-1 and γ-secretase pharmacological inhibitors both prevented the lyso-Gb3-induced upregulation of *HES1* and chemokines at the protein and mRNA levels, as well as the increase in NFκB DNA-binding activity, thereby curbing proinflammatory responses [110,119,120,121]. A summary model of the discussed pathways is provided in Figure 1.

### 3.2. The Sphingomyelinase-Pathway

Diabetic kidney disease (DKD) and focal segmental glomerulosclerosis (FSGS) have been tightly linked to podocyte dysfunction and can lead to end-stage renal disease (ESRD) and kidney failure. Recent studies have contributed to our understanding of the role that the lipid metabolizing enzyme sphingomyelinase phosphodiesterase acid-like 3b (SMPDL3b) plays in both of the mentioned diseases. SMPDL3b controls the activity of acid sphingomyelinase. In the podocytes, SMPDL3b is located in the membrane lipid rafts, and its role is hypothesized to be the conversion of sphingomyelin to ceramide and phosphorylcholine due to its homology with acid sphingomyelinase (ASMase), thus exerting signaling and regulatory roles. However, its exact intrinsic enzymatic function is yet to be determined [117,122,123,124,125].

DKD is the number one cause of renal failure in the USA and is most importantly characterized by the loss of podocytes, also known as podocytopenia, following their injury, in type 1 and type 2 diabetic patients [126,127,128,129,130]. Although high levels of sphingolipids have been reported in the plasma of diabetic individuals, recently it has become clear that it is the intracellular sphingolipid composition of glomerular cells, especially podocytes, that contributes to the pathogenesis of diabetic nephropathy [2,131,132,133]. Interestingly, SMPDL3b expression levels were higher in glomeruli of patients with DKD, those of diabetic mice and in cultured human podocytes treated with serum from DKD patients. Indeed, due to its theorized role, Fornoni et al. hypothesized that this increase in the expression of SMPDL3b will activate sphingomyelin metabolic pathways and lead to the accumulation of different sphingolipids in glomerular cells [134]. Taken together, studies conducted by Fornoni’s group indicate a substantial correlation between ceramide accumulation, glomerular hypertrophy and podocytopenia in DKD [134,135,136,137].

Focal segmental glomerulosclerosis (FSGS) is the most common cause of nephrotic syndrome and glomerular diseases in adults [138]. FSGS can be caused genetically by mutations in genes coding for podocyte proteins or non-genetically, taking primary spontaneous forms. Its recurrence is also very common post-transplantation in almost one-third of such patients [139,140,141]. Unlike in DKD, SMPDL3b is downregulated in FSGS patients. In a study by Fornoni’s group involving 41 patients with high risk of FSGS recurrence, SMPDL3b levels in podocytes were found to be decreased in patients in who FSGS recurred. This lead to the hypothesis that low levels of the SMPDL3b enzyme, leads to the accumulation of non-metabolized sphingomyelin that might play a role in FSGS pathogenesis. Indeed, this is verified by the observation that cultured podocytes treated with serum from FSGS patients have low activity of the ASMase enzyme in addition to decreased SMPDL3b expression levels. This was associated with actin cytoskeletal remodeling and apoptosis, both of which could be prevented by overexpressing SMPDL3b in the podocytes [124].

In a study aimed at exploring the dynamics of podocyte foot processes, the researchers investigated the role of urokinase plasminogen activator receptor (uPAR), a molecule known to be involved in cellular motility, during proteinuric kidney diseases [142,143,144]. In fact, uPAR plays remarkable roles in a variety of processes that are based on cell movement like stem cell mobilization and tumor metastasis. In FSGS patients, circulating soluble uPAR (suPAR) levels are elevated, leading to αVβ3 integrin activation in podocytes, a pathway hypothesized to contribute to the proteinuria observed in these individuals. The suPAR-dependent activation of αVβ3 in FSGS is linked to the downregulation of SMPDL3b and decreased RhoA kinase activity. Conversely, increased SMPDL3b levels were shown to prevent suPAR-αVβ3 interaction in DKD, thus preventing podocyte cytoskeleton remodeling and migration, while leading to an increase in RhoA kinase activity [124,134,142,145,146]. Podocytes are highly differentiated glomerular cells that depend mainly on the integrity of their cytoskeleton to properly contribute to the critical function of the kidney ultrafiltration barrier [147]. These results thus highlight the importance of SMPDL3b and hence sphingomyelin and its catabolites in regulating podocytes physiological functions and response in pathophysiological conditions and makes them a target for new strategies that can prevent or reverse podocyte injury in two common kidney diseases.

**Figure 2:**
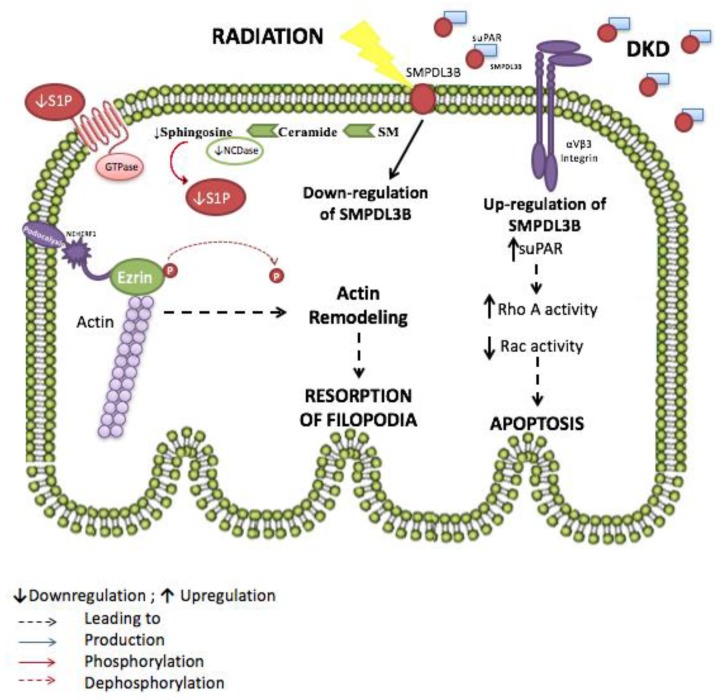
Proposed role for sphingomyelinase phosphodiesterase acid-like 3b (SMPDL3b) in radiation and diabetic kidney disease-induced podocytopathy. Radiation causes the downregulation of SMPDL3B, altering the sphingolipid metabolism. Ceramide levels increase while sphingosine and sphingosine 1 phosphate levels decrease. This leads to the dephosphorylation of ezrin and subsequent actin remodeling and resorption of filopodia. However, in diabetic kidney disease (DKD) SMPDL3B levels are elevated and bind to increased circulating soluble urokinase plasminogen activator receptor (suPAR), thus inhibiting the activation of αVβ3, but enabling RhoA activation and increasing podocyte apoptosis.

In fact, the observations described earlier correlate with the findings of our recent work aiming to study the molecular mechanisms involved in radiation-induced podocyte injury and radiation nephropathy. Exposure to radiation during abdominal or paraspinal cancer treatment usually affects different kidney components and the damage induced is proportional to the dosage and duration of radiotherapy. However, excessive exposure can lead to a syndrome of kidney failure called radiation nephropathy, which is, interestingly, a secondary cause for FSGS. This condition clinically manifests itself by proteinuria and reduced glomerular filtration rate and eventually leads to end-stage renal disease (ESRD), hence requiring either dialysis or transplantation [148,149,150,151,152]. In order to assess whether SMPDL3b plays a role in radiation-induced podocytopathy, podocytes were exposed to increasing doses of radiation. Within two hours after radiation, cultured human podocytes exhibited remarkable morphological changes. The actin-binding protein ezrin translocated from the cell periphery, where it stabilizes the link between the actin filaments and the plasma membrane, into the cytosol, resulting in cytoskeleton remodeling. Also, radiation led to decreased SMPDL3b protein levels, whereas overexpressing SMPDL3b protected podocytes from post-radiation ezrin relocation, cytoskeleton remodeling and apoptosis. Interestingly, when investigating cellular sphingolipid levels, an increase in ceramide levels after irradiation was observed in all the podocytes except those overexpressing SMPDL3b. In addition, a time-dependent drop in neutral ceramidase activity and in sphingosine and S1P levels were observed. The administration of exogenous S1P mitigated the radiation-induced cytoskeletal changes, thus proving the protective role of S1P against radiation injury. The proposed mechanism to explain these changes is that radiation induces downregulation of SMPDL3b thus altering sphingolipid metabolism by raising ceramide levels and decreasing sphingosine and S1P. This leads to a marked dysregulation of the cytoskeletal network within the podocyte, ultimately causing cellular injury which explains the observed clinical phenotype [153] (Figure 2).

Cardiovascular morbidities are a leading cause of death in patients with end-stage renal disease (ESRD) [154]. Indeed, patients on dialysis and those who have received a kidney transplant have a much higher rate of cardiac diseases as compared to the general population. These include fatal ischemic heart disease, atherothrombotic vascular disease and myocardial infarctions [155,156,157]. However, since vascular risks alone do not explain the elevated cardiovascular mortality in renal patients, hyperhomocysteinemia has been suspected to play a role. In fact, studies support the presence of a physiological link between blood homocysteine levels and heart diseases [158,159]. Homocysteine is the transmethylation metabolite of the essential amino acid methionine. This product is present in different forms in the plasma, the fasting level of most of which is increased in renal patients, even with mild degrees of renal insufficiency, leading to a pathological condition known as hyperhomocysteinemia (hHcys) [160,161,162]. Excess homocysteine in hyperhomocysteinemia is cardiotoxic since it can cause lipid oxidation, impaired thrombolysis and vascular smooth muscle differentiation, as well as vasodilation and monocyte chemotaxis, all of which predispose for severe cardiovascular complications [158,163,164].

The role of the sphingomyelinase pathway has been investigated in hyperhomocysteinemia. Boini and colleagues first assessed levels of homocysteine in wild-type mice and in mice where ASMase expression was either silenced by a short hairpin RNA (shRNA) or knocked out (*ASMase*^−/−.^) No difference was observed in the plasma levels of homocysteine in both models thus affirming that ASMase does not play a role in the pathophysiology of hyperhomocysteinemia. However, ASMase gene deficiency lead to noticeable results when such mice were fed a folate-free diet, a diet known to retard methionine metabolism, thereby inducing hyperhomocysteinemia. *ASMase*^+/+^ mice showed increased levels of ceramide, ASMase mRNA production and activity and urinary proteinuria. Local oxidative stress was also observed in these mice through the production of oxygen free-radicals (O_2_^−.^) by NADPH (nicotinamide adenine dinucleotide phosphate) oxidases in the cortex of the glomeruli and in the podocytes, thus leading to glomerular damage. Many studies that were conducted using pharmacological and/or molecular interventions have in fact reported that the observed activation of NADPH oxidase occurs due to the ceramide produced by the action of hyperhomocysteinemia [165,166,167,168]. However, *ASMase*^−/−^ mice had significantly lower levels of ceramide, ASMase activity and O_2_^−^· production showing that the glomerulus was protected from oxidative stress and injury [169]. This indicates an active role of the sphingomyelinase pathway in hyperhomocysteinemia-induced renal injury and stress.

The role of ASMase in hHcys was further studied using the cystathionine β-synthase (Cbs) gene and *ASMase* gene double knockout mice model. *Cbs* gene knockout mice *(Cbs*^+/−^) are known to develop hHcys characterized by glomerular oxidative stress and podocyte injury. The results showed that *ASMase*^+/+^ and *ASMase*^−/−^ homozygous mice, and heterozygous mice with ASMase^+/−^, have the same blood Hcys levels in the same *Cbs*^+/+^ background. However, hHcys itself increases ASMase activity and renal ceramide levels, mainly in the podocytes, in *Cbs*^+/−^/*ASMase*^+/+^ and *Cbs*^+/−^/*ASMase*^+/−^ mice, which is accompanied by oxidative stress, glomerular damage and proteinuria. This enzymatic activity can be attenuated in *ASMase*^−/−^ mice with hHcys, thus reducing podocyte ceramide accumulation and curbing the observed renal complications. Therefore, the sphingomyelinase pathway plays a pathogenic role in hHcys and its blockade can be protective against hHcys-inducted renal nephropathy [125].

### 3.3. Sphingosine-1-Phosphate

Sphingosine-1-phosphate (S1P), a sphingolipid produced intracellularly in organelles and the plasma membrane through multi-step metabolic pathways, is also involved in regulating and maintaining normal podocyte function [170,171,172,173]. S1P acts via G-protein coupled receptors. Five S1P receptors (S1PRs) have been identified to date (S1PR1 to S1PR5) but only receptors 1–3 have been described in the kidney [153]. S1PRs are thought to be involved in multiple critical cellular and physiological processes including cell survival, proliferation, differentiation, secretion and migration and immune responses [54,174,175,176,177,178,179,180]. Hence, the physiological concentration of S1P is actively maintained within an optimal narrow range through the coordinating activities of biosynthetic and biodegradative enzymes [173,181,182].

In radiation-induced podocyte injury, S1P plays a renal protective effect. In fact, the treatment of irradiated podocytes with exogenous S1P attenuated cytoskeletal remodeling post-radiation, suggesting that S1PRs play a critical role in maintaining the podocytes integrity upon stress and hence their role in the ultrafiltration barrier [153]. In addition, work done by Awad and colleagues demonstrated that independent S1PR1 activation using selective and non-selective S1PR1 agonists such as SEW2871 and FTY720 reduces early stage diabetic nephropathy. The levels of both albumin and tumor necrosis factor α (TNF-α) in the urine of diabetic rats dropped significantly upon treatment with the mentioned agonists [183]. S1PR1 agonists have also been noted to play a role in mitigating renal injury due to ischemia reperfusion [184].

A recent study conducted by Schuman et al. investigated the role of S1P lyase in the kidney, among other tissues. Sphingosine-1-phosphate lyase (SGPL1) is an intracellular microsomal enzyme expressed to varying degrees in almost all mammalian cells and carries a critical irreversible step in sphingolipid metabolism by degrading S1P. Prasad et al. showed that loss of function mutation in the gene coding this decisive enzyme can lead to a wide array of systemic problems. In fact, a partial or total lack of S1P lyase leads to the accumulation of S1P in body tissues and disturbs the established S1P concentration gradient between blood and lymphatic tissues, hindering normal immune-related physiological processes [185,186,187,188,189,190,191,192,193,194]. Interestingly though, current research supports the potential use of S1P lyase inhibition as a treatment option for various autoimmune disorders [192,193,195]. To assess the safety of this approach, a tamoxifen-inducible partial S1P lyase-deficient mice were generated. Kidney alterations were observed in mice that are partially S1P lyase deficient upon induction of SRBC-induced DTH (sheep red blood cells-induced delayed-type hypersensitivity), including the matrix deposition and destruction of some capillaries, both of which are characteristics of glumerulopathy. Also, S1P lyase-deficient mice, in which DTH was not experimentally induced, revealed podocyte foot processes effacement associated with a decrease in serum protein and albumin levels and an increase in urine protein-creatinine levels, characteristics of glomerular injury, podocytopathy and proteinuria. This was accompanied by a remarkable increase in S1P levels in the kidney. Taken together, these results suggest that S1P accumulation due to S1P lyase deficiency causes renal damage and podocyte dysfunction. The mice were housed ad libitum and followed for over a year. The mortality rate in the partially S1P lyase-deficient mice was much higher than in the control mice. The animals who survived till the end of experimental period showed severe renal damage upon histopathological examination. Kidney analysis revealed glomerular lesions, focal and segmental glomerulosclerosis, interstitial fibrosis and tubular degeneration. This was accompanied by chronic proteinuria. It is important to note that those pathological alterations linked to reduction in S1P lyase activity are obtained in partially deficient mice due to the lethality of a totally deficient phenotype. The reduction of S1P lyase levels caused, in addition to podocyte-based kidney toxicity, manifestation of skin irritation and platelet activation in the studied mice, putting in question the safety of treating systematic chronic autoimmune diseases with S1P lyase inhibitors [58].

Steroid-resistant nephrotic syndrome (SRNS) is a congenital condition that often presents with a set of serious comorbidities including adrenal insufficiency, pathological skin conditions and/or neurological defects [59]. A recent study of seven families affected by SRNS, nine different mutations were identified, upon whole exome sequencing (WES), in the gene coding for SGPL1. SRNS is a serious condition which, when accompanied with resistance to other immunosuppressive reagents, can lead to kidney failure. It is responsible for 15% of all end-stage renal disease cases that manifest in individuals younger than 25 years old [196,197]. Further investigations revealed focal segmental glomerulosclerosis at the kidney level. Knocking down SGPL1 in rat mesangial cells inhibited their normal physiological functions. The obtained abnormal phenotype was partially corrected through using specific S1PR1 antagonists. In addition, mutant flies that lacked SGPL1 enzymatic activity demonstrated a nephrotic syndrome-like phenotype, which is equivalent to podocyte injury in humans [59]. Hence, S1P imbalance is indeed implicated in injurious nephrotic diseases, among other complications, further affirming that potential therapeutic role of S1P lyase inhibitors.

## 4. Sphingolipids as Potential Therapeutic Targets

In the light of the important role of the sphingolipid metabolism in maintaining podocyte and glomerular integrity, recent studies have focused on these bioactive lipids as potential therapeutic targets for various nephropathic diseases.

Focal segmental glomerulosclerosis and diabetic kidney disease are marked by proteinuria and are a major cause for progressive end-stage renal disease. In this context, SMPDL3b has been recently reported to be downregulated in FSGS but increased in DKD. The hypothesis postulated was that targeting SMPDL3b and regulating its expression levels would indeed protect the podocytes from damage [124,134].

Rituximab is a genetically engineered dimeric monoclonal antibody that is designed to target CD20 expressed on B lymphocytes [198], and happens to bind to SMPDL3b through a specific amino acid sequence [199]. It is currently used as a therapy for different immune diseases and has been beneficial in multiple kidney diseases such as steroid resistant nephrotic syndrome and acute allograph rejections [200]. Earlier findings revealed that treating kidney transplant patients with rituximab reduces the incidence of post-transplant proteinuria, and leads to a decrease in the estimated glomerular filtration rate (GFR). Interestingly, patients who redeveloped FSGS showed a lower number of SMPDL3b-expressing podocytes [124].

Our recent work showed that podocyte SMPDL3b levels are downregulated after radiation, leading to actin remodeling and the resorption of filopodia. However, the overexpression of SMPDL3b mitigated these changes [153]. Pre-treatment with rituximab inhibited SMPDL3b downregulation and, thus, had a similar effect to SMPDL3b overexpression. In fact, radiation-induced cytoskeletal changes were not observed and caspase-3 cleavage was attenuated in the pre-treated podocytes [153]. These findings place rituximab in a clinician’s perspective as a potential protective agent against radiation-induced kidney injury and not only as an anti-tumor agent.

Another therapy targeting sphingolipids is enzyme replacement therapy (ERT). ERT presents the lacking enzyme to the body of the patient, not correcting the initial cause of the disease but delivering the right amount of the enzyme that would make up for its deficiency [201]. Different studies have demonstrated the beneficial effect of ERT in reducing glycosphingolipid lysosomal accumulation. ERT with α galactosidase A was found to help clear Gb3 accumulation in Fabry’s disease patients. In an ERT study that extended over a period of 11 months of a population of 58 Fabry’s disease patients, renal biopsies revealed that nearly all cell types of the kidney had cleared Gb3 accumulation, especially in podocytes and distal tubular epithelium [202]. A more recent study showed that ERT with agalsidase β leads to a significant decrease of 73% of Gb3 accumulation from the podocytes after one year of treatment [203]. In addition, prolonged ERT with agalsidase reduced Gb3 inclusions in podocytes and microalbuminuria in Fabry’s patients, thus advocating for a protective role of ERT on early nephropathy [204].

**Figure 3:**
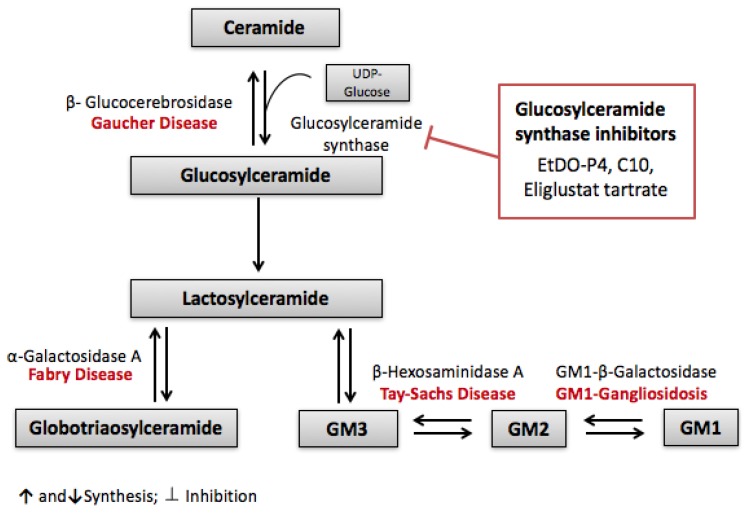
Substrate reduction theory: Glucosylceramide synthase inhibitors are therapeutic targets for lipid storing diseases. Glucosylceramide synthase inhibitors (EtDO-P4, C10 and Eliglustat tartrate) halt the synthesis of glucosylceramide, thereby preventing the downstream synthesis and accumulation of different sphingolipids involved in diseases such as Gaucher, Fabry’s, Tay-Sachs and GM1-Gangliosidosis.

Although ERT is a very promising treatment for lipid storage disease, it alone is not able to reverse all the damage induced by these diseases. Substrate reduction theory, postulated by Norman Radin, is another possible therapeutic approach for lipid storage diseases [205]. Substrate reduction theory is based on reducing the accumulation of lipids by inhibiting their synthesis rather than correcting the initial enzymatic dysfunction. Slowing the biosynthesis of the accumulating lipid by synthesis inhibition would balance the rate of hydrolysis, thus preventing its accumulation and the resulting complications [206]. The substrate reduction theory was first carried on the lipid storage disease Gaucher disease type 1. Gaucher disease is a recessive autosomal disease characterized by a loss or lack of activity in β-glucocerebrosidase, leading to the accumulation of the sphingolipid glucosylceramide in lysosomes [207]. This glucosylceramide synthase is responsible for the synthesis of glucosylceramide from ceramide and UDP-glucose, a critical step in glucocerebroside-based glycosphingolipid pathway [208]. Further products of this pathway are globotriaosylceramide and gangliosides, as seen in Figure 3. These glycosphingolipids are involved in the pathology of even other lipid storage diseases such as Fabry’s, Tay Sachs and Gangliosidosis. Inhibiting the first step of this pathway therefore became a potential therapeutic target for these diseases replacing or complementing enzyme replacement therapy. PDMP was the first glucosylceramide synthase inhibitor designed and was found to be effective in reducing lipid accumulation over different cell lines and animal models of Fabry’s disease [205]. Even better, a derivative of PDMP called EtDO-P4 was tested on Fabry’s disease cellular models. As early as three days of treatment, approximately 70% of globotriaosylceramide was depleted [209]. A model of black C57BL/6 Fabry’s disease mice also showed a concentration-dependent decrease of glucosylceramide accumulation in kidney, liver and spleen after treatment with EtDO-P4 [210]. However, both Fabry’s and Gaucher disease exhibit accumulations of lipids in the CNS (central nervous system) involving neurological symptoms and leading to premature death, and since the mentioned pharmaceutical chemicals cannot cross the blood brain barrier [211], other glucosylceramide synthase inhibitors are currently in clinical development [212].

Targeting glucosylceramide synthesis has been studied in renal injury related to chemotherapy. Cisplatin, a widely used chemotherapeutic agent for solid tumors, is associated with acute kidney injury (AKI). Both ceramide and glucosylceramides were found to play a role in cisplatin-induced kidney injury. Treating mice with cisplatin elevated the levels of both ceramide and glucosylceramide in the renal cortex, leading to the activation of inflammatory responses, oxidative stress and apoptosis. The co-administration of Amitriptyline, an inhibitor of acid sphingomyelinase, and Myriocin, an inhibitor of the de novo ceramide synthetic pathway, prevented the accumulation of ceramide in the renal cortex. However, inhibiting the conversion of ceramide into glucosylceramide using C10, a GCS inhibitor, exacerbated cisplatin-induced renal injury, suggesting that glucosylceramide has a protective role and acts as buffer in order to attenuate the effect of ceramide accumulation [213] (Figure 3).

## 5. Conslusions

In addition to cancer biology and inflammation, sphingolipids are evolving as key players in renal disorders. Recent studies identify SMPDL3b, GCS, S1P lyase and SK as key targets within sphingolipid metabolism for developing novel therapies against renal disorders. Several sphigolipid analogues, recombinant enzymes , and their inhibitors are expected to enter clinical trials aimed at treating various nephropathies, in the near future.
